# Dose invasive apocrine adenocarcinoma has worse prognosis than invasive ductal carcinoma of breast: evidence from SEER database

**DOI:** 10.18632/oncotarget.15597

**Published:** 2017-02-21

**Authors:** Ning Zhang, Hanwen Zhang, Tong Chen, Qifeng Yang

**Affiliations:** ^1^ Department of Breast Surgery, Qilu Hospital, Shandong University, Jinan, 250012, Shandong Province, People's Republic of China; ^2^ Pathology Tissue Bank, Qilu Hospital, Shandong University, Jinan, 250012, Shandong Province, People's Republic of China

**Keywords:** apocrine adenocarcinoma, breast cancer, surveillance, epidemiology and end result (SEER), prognosis

## Abstract

**Background:**

Invasive apocrine adenocarcinoma (AAC) of breast is a rare histopathological subtype of breast carcinomas. We aim to investigate the different characteristics and prognostic outcomes between AAC and invasive ductal carcinoma (IDC) of breast cancer.

**RESULTS:**

AAC patients presented with older ages, more aggressive behaviors, lower ER and PR proportions, higher HER2 amplification rates and less application of breast-conserving therapy and adjuvant chemotherapy compared to IDC patients. Long-term OS and DSS were both worse in ACC patients (*p* = 0.006, *p* = 0.012 respectively) than in IDC patients by Kaplan-Meier analysis. However, no significant difference was detected in DSS (*p* = 0.181) and OS (*p =* 0.116) between the matched two histological subtypes. Further subgroup analysis indicated that AJCC stage, ER status, PR status and HER2 status may be principal confounders for AAC prognosis.

**Materials and Methods:**

With accession to the Surveillance, Epidemiology and End Result (SEER) database, a total of 260,596 patients met the eligibility criteria. Clinicopathological characteristics were compared between groups using Chi-square test. Univariate and multivariate analyses were applied to evaluate the overall survival (OS) and disease-specific survival (DSS). Subgroup analyses summarized the hazard ratio (HR) of AAC versus IDC using a forest plot.

**Conclusions:**

AAC had unique clinicopathological characteristics and it tended to be a more aggressive type than IDC. However, the worse prognosis was diminished after matching for demographic and clinicopathological factors. Deeper insights into AAC are in need to contribute to individualized and tailored therapy, which thereby may improve clinical management and outcomes.

## INTRODUCTION

Invasive apocrine adenocarcinoma (AAC) of breast is a rare histopathologically defined subtype of breast carcinoma, which is morphologically characterized by abundant eosinophilic and granular cytoplasm, large nuclei with prominent nucleoli, and distinctive cell membrane by hematoxylin and eosin (H&E) staining [1]. Furthermore, they tend to exhibit a characteristic hormone receptor profile: estrogen receptor (ER)-negative, progesterone receptor (PR)-negative and androgen receptor (AR)-positive, either human epidermal growth factor receptor-2 (HER2)-positive or (epidermal growth factor receptor) EGFR-positive [1–3]. Although the immunohistochemical characteristics are helpful for the proper recognition of the apocrine carcinomas, the presence of malignant apocrine cells in more than 90% of the tumor population strictly defines AAC of breast [4].

According to reported data, invasive ductal carcinoma (IDC, non-specific type, NST) and invasive lobular carcinoma account for about 75% and 15% of all invasive breast carcinoma, respectively [5]. Nevertheless, AAC constitutes between 0.3 and 4% of invasive breast carcinoma [4, 6–8]. Due to rarity of this entity of breast cancer, clinicopathological characteristics and prognoses of patients with AAC were only reported in limited number of studies: either case reports or studies recruiting a small number of patients. Consequently, the available data are sometimes contradictory. In addition, the prognostic values of demographic and clinicopathological characteristics in AAC therefore remain unclear. Matsuo et al. reported patients with AAC were older than those with IDC [9]. However, they only involved 12 patients in their study. Tanaka et al. found lower frequency of axillary nodal involvement in AAC compared to IDC [10]; while Dreyer et al. showed 7 out of 14 apocrine breast carcinomas with positive lymph node status [11]. With respect to prognosis, some of these studies indicated better prognosis in AAC than IDC patients [12–15]; while in some other studies, no significant difference or even poor survival outcome of AAC was detected compared to non-AAC [7, 10, 16–20]. Considering the absence of comprehensive understanding of AAC, AAC managements are currently based on evidence from studies of IDC, which sometimes may be inappropriate. Identifying the prognostic factors of AAC would help to acquire a better knowledge of the disease and make better therapeutic guidelines. Therefore, it is of great importance to clarify the clinicopathological characteristics and prognostic factors of AAC in a large population.

To gain better knowledge of clinicopathological characteristics and prognostic differences between AAC and IDC, we conducted the present study utilizing the Surveillance, Epidemiology, and End Results (SEER) database. In all, 840 patients with AAC were collected in our study, which contained the largest number of AAC patients compared to other published studies to our best knowledge. We aimed at determining the prognostic factors that may account for survival differences between these two histological subtypes of breast cancer.

## RESULTS

### Demographics and clinical characteristics of study population

A total of 260,596 patients met the eligibility criteria for our study, including 840 (0.32%) AAC patients and 259,756 (99.68%) IDC patients. The demographics, tumor and treatment type characteristics were summarized and compared between the two cohorts in Table 1. Significant differences were found in demographics including age and race, tumor characteristics including grade, tumor size, LN status, AJCC stage, ER status, PR status and HER2 status and treatment including surgery type and radiation by comparing the two histological subtypes. AAC patients showed an older age at diagnosis (50–79 years, 81.1% vs. 70.9%, respectively; *p <* 0.001) and tended to have a significantly lower proportion of white race (75.6% vs. 79.1%, respectively; *p* = 0.013) than IDC patients. AAC patients presented more frequently with larger tumors (tumor size > 5 cm, 7.4% vs. 5.0%, respectively; *p <* 0.001) and more grade II and III+UD tumors, namely poorly differentiated tumors (grade II: 46.4% vs. 39.3%; grade III+UD: 43.2% vs. 39.3%, respectively; *p <* 0.001). In addition, the rate of positive LN in AAC patients is higher than that in IDC patients (37.4% vs. 32.3%, respectively; *p* = 0.001). Collectively, it comes naturally that AJCC stage III patients account for higher proportion in AAC patients than in IDC patients (17.6% vs. 12.3%, respectively; *p <* 0.001). A larger proportion of AAC patients were detected with negative ER status (70.1% vs. 22.8%, respectively; *p <* 0.001) and negative PR status (76.8% vs. 32.9%, respectively; *p <* 0.001) than IDC patients. However, morepositive HER2 (7.6 vs. 6.8%, respectively; *p <* 0.001) status was shown in AAC patients than in IDC patients. Treatment also diverged between both groups. Breast conservation surgery (BCS) and adjuvant radiotherapy were less often applied on AAC patients than IDC patients (55.0% vs. 60.1%, respectively; *p* = 0.011. 52.4% vs. 56.9%, respectively; *p* = 0.026).

**Table 1:** Characteristics of patients from the SEER database by histologic subtype, AAC vs IDC

	AAC, *n* = 840	IDC, *n* = 259,756	Total, *n* = 260,596	*P*-Value^a^
	(%)	(%)	(%)	
Median follow-up (months) (IQR)	61 (31.25–94)	50 (23–83)	50 (23–83)	
Age at diagnosis (years)				
18–49	159 (18.9)	75,714 (29.1)	75,873 (29.1)	< 0.001
50–79	681 (81.1)	184,042 (70.9)	184,723 (70.9)	
Race				
White	635 (75.6)	205,447 (79.1)	206,082 (79.1)	0.013
Black	99 (11.8)	28,295 (10.9)	28,394 (10.9)	
Others^b^	104 (12.4)	24,625 (9.5)	24,729 (9.5)	
Unknown	2 (0.2)	1,389 (0.5)	1,391 (0.5)	
Marital status				
Married	496 (59.0)	156,646 (60.3)	157,142 (60.3)	0.131
Not married^c^	321 (38.2)	93,185 (35.9)	93,506 (35.9)	
Unknown	23 (2.7)	9,925 (3.8)	9,948 (3.8)	
Laterality				
Left	434 (51.7)	131,609 (50.7)	132,043 (50.7)	0.810
Right	406 (48.3)	128,119 (49.3)	128,525 (49.3)	
Only one side, NOS	0 (0.0)	28 (0.0)	28 (0.0)	
Grade				
I	59 (7.0)	48,702 (18.7)	48,761 (18.7)	< 0.001
II	390 (46.4)	102,176 (39.3)	102,566 (39.3)	
III + UD^d^	363 (43.2)	102,015 (39.3)	102,378 (39.3)	
Unknown	28 (3.3)	6,863 (2.6)	6,891 (2.6)	
Tumor size (cm)				
≤ 2	483 (57.5)	165,658 (63.8)	166,141 (63.8)	< 0.001
> 2 and ≤ 5	285 (33.9)	79,527 (30.6)	79,812 (30.6)	
> 5	62 (7.4)	13,053 (5.0)	13,115 (5.0)	
Unknown	10 (1.2)	1,518 (0.6)	1,528 (0.6)	
LN status				
Negative	497 (59.2)	169,043 (65.1)	169,540 (65.1)	0.001
Positive	314 (37.4)	83,935 (32.3)	84,249 (32.3)	
Unknown	29 (3.5)	6,778 (2.6)	6,807 (2.6)	
AJCC stage				
I	380 (45.2)	130,690 (50.3)	131,070 (50.3)	< 0.001
II	312 (37.1)	97,082 (37.4)	97,394 (37.4)	
III	148 (17.6)	31,984 (12.3)	32,132 (12.3)	
ER status				
Negative	589 (70.1)	59,323 (22.8)	59,912 (23.0)	< 0.001
Positive	251 (29.9)	200,433 (77.2)	200,684 (77.0)	
PR status				
Negative	645 (76.8)	85,524 (32.9)	86,169 (33.1)	< 0.001
Positive	195 (23.2)	174,232 (67.1)	174,427 (66.9)	
HER2 status				
Negative	187 (22.3)	86,291 (33.2)	86,478 (33.2)	< 0.001
Positive	64 (7.6)	17,740 (6.8)	17,804 (6.8)	
Borderline	7 (0.8)	2,362 (0.9)	2,369 (0.9)	
Unknown	582 (69.3)	153,363 (59.1)	153,945 (59.1)	
Surgery type				
Mastectomy	377 (44.9)	103,403 (39.8)	103,780 (39.8)	0.011
BCS	462 (55.0)	156,053 (60.1)	156,515 (60.1)	
Unknown	1 (0.1)	300 (0.1)	301 (0.1)	
Radiation				
No	365 (43.5)	103,177 (39.7)	103,542 (39.7)	0.026
Yes	440 (52.4)	147,735 (56.9)	148,175 (56.9)	
Unknown	35 (4.2)	8,844 (3.4)	8,879 (3.4)	

AJCC = American Joint Committee on Cancer, BCS = breast conserving surgery, ER=estrogen receptor, PR=progesterone receptor, HER2 = human epidermal growth factor receptor 2, IDC = infiltrating ductal carcinoma, IQR=interquartile range, LN = lymph node, NOS = no other specific, UD = undifferentiated, SEER = Surveillance, Epidemiology and End Results.

^a^*P*-value of the Chi-square test comparing the AAC and IDC groups.

^b^Including American Indian/Alaskan native, and Asian/Pacific Islander, and others-unspecified.

^c^Including divorced, separated, single (never married), unmarried, domestic partner and widowed.

^d^Including grade 3 and undifferentiated.

### Comparison of survival between IDC and AAC patients

**Figure 1:**
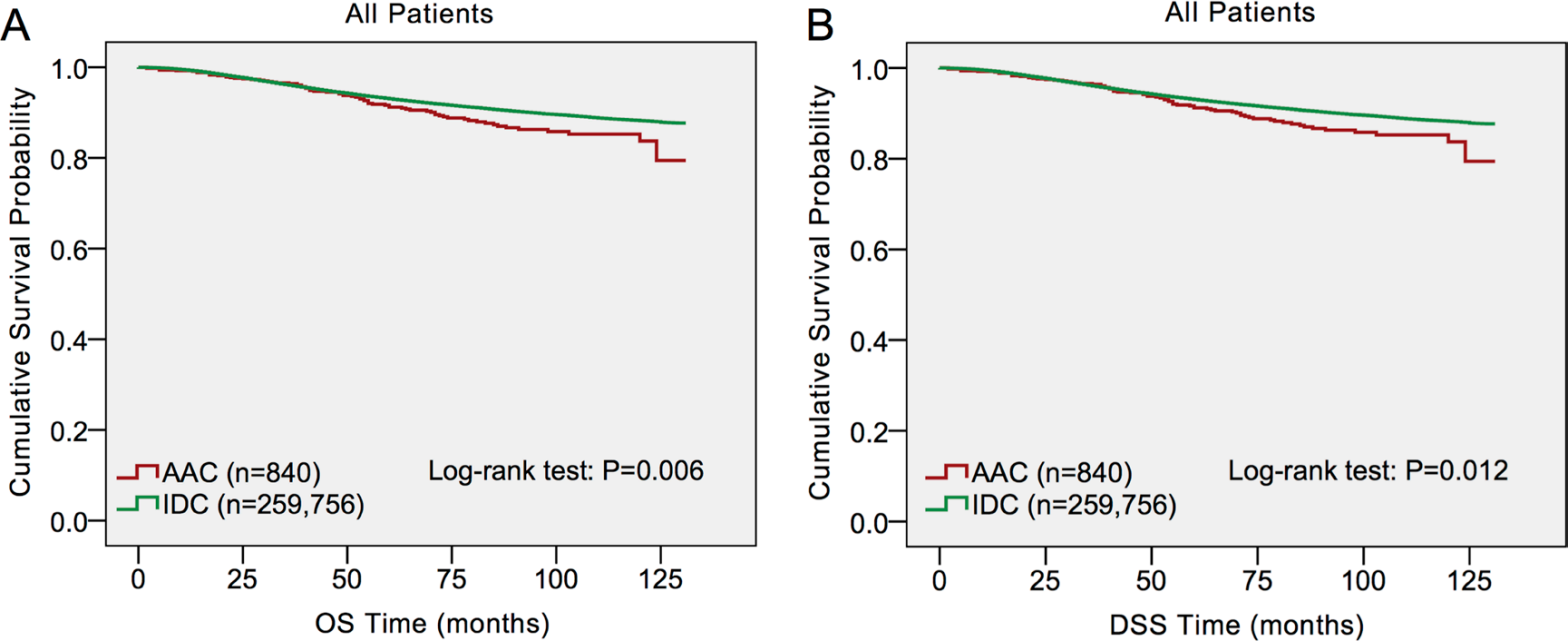
Log-rank test for breast cancer overall survival (OS) and disease-specific survival (DSS) to compare invasive apocrine adenocarcinoma (AAC) to infiltrating ductal carcinoma (IDC)

Kaplan–Meier plots were used to evaluate overall survival (OS) and disease-specific survival (DSS) in these two histological subtypes (Figure 1). As the plots illustrated, OS and DSS were both worse in ACC patients (*p* = 0.006, *p* = 0.012 respectively) than in IDC patients. In order to further investigate the effects of prognostic factors of OS and DSS, a multivariate analysis by Cox proportional hazards model was performed (Table 2). For both DSS and OS, the multivariate analysis validated that older age at diagnosis, black race, not married status, grade II /III and UD, tumor size > 2 cm, positive lymph node status were associated with poor outcomes, while ER positivity and PR positivity, BCS and radiation were protective factors for DSS. However, AAC histology was found not to be an independent prognostic factor after multivariate analysis in Cox proportional hazard model (AAC vs. IDC, HR = 0.834, 95% CI = 0.695–1.002, *p* = 0.052 (OS); HR = 0.800, 95% CI = 0.639–1.001, *p* = 0.051 (DSS); respectively).

**Table 2:** Multivariate analysis of overall survival (OS) and disease-specific survival (DSS) predictors using cox proportional hazard model

	OS		DSS	
	HR (95% CI)	*P*-Value^a^	HR (95% CI)	*P*-Value^a^
Age at diagnosis (years)				
18–49	Reference	-	Reference	-
50–79	1.663 (1.615–1.713)	< 0.001	1.199 (1.160–1.240)	< 0.001
Race				
White	Reference	-	Reference	-
Black	1.276 (1.233–1.321)	< 0.001	1.293 (1.240–1.348)	< 0.001
Others^b^	0.756 (0.719–0.796)	< 0.001	0.778 (0.732–0.828)	< 0.001
Unknown	0.353 (0.257–0.486)	< 0.001	0.399 (0.273–0.582)	< 0.001
Marital status				
Married	Reference	-	Reference	-
Not married^c^	1.460 (1.422–1.499)	< 0.001	1.241(1.201–1.282)	< 0.001
Unknown	1.119 (1.039–1.207)	0.003	1.004 (0.913–1.105)	0.928
Histology type				
IDC	Reference	-	Reference	-
AAC	0.834 (0.695–1.002)	0.052	0.800 (0.639–1.001)	0.051
Grade				
I	Reference	-	Reference	-
II	1.252 (1.191–1.316)	< 0.001	2.257 (2.052–2.483)	< 0.001
III + UD^d^	1.671 (1.588–1.758)	< 0.001	3.685 (3.351–4.051)	< 0.001
Unknown	1.161 (1.053–1.280)	0.003	2.266 (1.964–2.615)	< 0.001
Tumor size (cm)				
≤ 2	Reference	-	Reference	-
> 2 and ≤ 5	1.741 (1.690–1.794)	< 0.001	2.190 (2.106–2.278)	< 0.001
> 5	2.994 (2.864–3.131)	< 0.001	3.891 (3.690–4.103)	< 0.001
Unknown	4.007 (3.687–4.355)	< 0.001	5.578 (5.089–6.114)	< 0.001
LN status				
Negative	Reference	-	Reference	-
Positive	2.014 (1.958–2.072)	< 0.001	2.775 (2.675–2.878)	< 0.001
Unknown	3.148 (2.967–3.339)	< 0.001	3.198 (2.940–3.478)	< 0.001
ER status				
Negative	Reference	-	Reference	-
Positive	0.743 (0.714–0.772)	< 0.001	0.683 (0.652–0.716)	< 0.001
PR status				
Negative	Reference	-	Reference	-
Positive	0.771 (0.743–0.801)	< 0.001	0.641 (0.611–0.672)	< 0.001
Surgery type				
Mastectomy	Reference	-	Reference	-
BCS	0.832 (0.807–0.857)	< 0.001	0.794 (0.765–0.824)	< 0.001
Unknown	1.088 (0.828–1.428)	0.546	1.517 (1.138–2.022)	0.005
Radiation				
No	Reference	-	Reference	-
Yes	0.779 (0.757–0.802)	< 0.001	0.872 (0.842–0.903)	< 0.001
Unknown	0.942 (0.877–1.011)	0.096	1.012 (0.930–1.102)	0.778

AAC = apocrine adenocarcinoma, IDC = infiltrating ductal carcinoma, ER = estrogen receptor, PR = progesterone receptor, HER2 = human epidermal growth factor receptor 2, LN = lymph node, NOS = no other specific, BCS = breast conserving surgery, HR = hazard ratio, CI = confidence interval, DSS = disease-specific survival, OS = overall survival.

^a^*P*-value was determined by multivariate Cox proportional hazard regression model.

^b^Including American Indian/Alaskan native, and Asian/Pacific Islander, and others-unspecified.

^c^Including divorced, separated, single (never married), unmarried, domestic partner and widowed.

^d^Including grade 3 and undifferentiated.

### Survival analysis in matched groups

**Figure 2:**
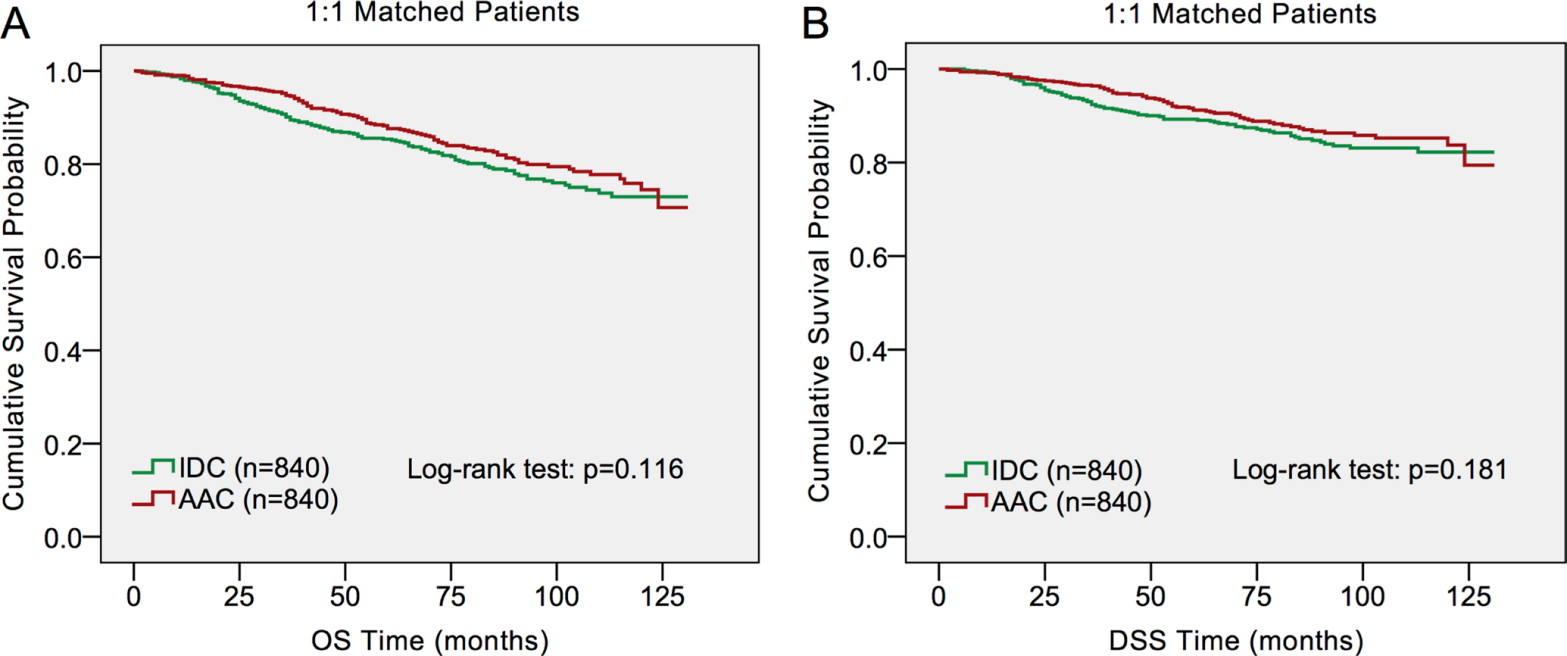
Log-rank test of 1:1 matched groups to compare invasive apocrineadenocarcinoma (AAC) to infiltrating ductal carcinoma (IDC)

To ensure that baseline differences in demographics and clinical characteristics across the two histological subtypes do not account for the outcome discrepancies, we carried out a 1:1 (IDC/AAC) matched case-control analysis using the propensity score matching method. A group of 1,680 patients were obtained, including 840 patients for each histological type (Table 3). In the matched groups, only tumor grade (*p <* 0.001) and tumor size (*p* = 0.034) were significantly different between AAC patients and IDC patients. Furthermore, no significant difference was detected in OS (*p* = 0.116) and DSS (*p* = 0.181) between the two histological subtypes (Figure 2).

**Table 3:** Characteristics of patients by histology subtype in 1:1 matched, AAC versus IDC

	AAC, *n* = 840	IDC, *n* = 840	Total, *n* = 1,680	*P*-Value^a^
	(%)	(%)	(%)	
Median follow-up (months) (IQR)	61 (31.25–94)	60 (29–92)	61 (30–93)	
Age at diagnosis (years)				
18–49	159 (18.9)	151 (18.0)	310 (18.4)	0.615
50–79	681 (81.1)	689 (82.0)	1,370 (81.6)	
Race				
White	635 (75.6)	613 (73.0)	1,248 (74.3)	0.199
Black	99 (11.8)	124 (14.8)	223 (13.3)	
Others^b^	104 (12.4)	98 (11.7)	202 (12.0)	
Unknown	2 (0.2)	5 (0.6)	7 (0.4)	
Marital status				
Married	496 (59.0)	504 (60.0)	1,000 (59.5)	0.368
Not married^c^	321 (38.2)	304 (36.2)	625 (37.2)	
Unknown	23 (2.70)	32 (3.80)	55 (3.3)	
Laterality				
Left	434 (51.7)	410 (48.8)	844 (50.2)	0.314
Right	406 (48.3)	429 (51.1)	835 (49.7)	
Only one side, NOS	0 (0.0)	1 (0.1)	1 (0.1)	
Grade				
I	59 (7.0)	81 (9.6)	140 (8.3)	< 0.001
II	390 (46.4)	313 (37.3)	703 (41.8)	
III + UD^d^	363 (43.2)	433 (51.5)	796 (47.4)	
Unknown	28 (3.3)	13 (1.5)	41 (2.4)	
Tumor size (cm)				
≤ 2	483 (57.5)	511 (60.8)	994 (59.2)	0.034
> 2 and ≤ 5	285 (33.9)	270 (32.1)	555 (33.0)	
> 5	62 (7.4)	58 (6.9)	120 (7.1)	
Unknown	10 (1.2)	1 (0.1)	11 (0.7)	
LN status				
Negative	497 (59.2)	496 (59.0)	993 (59.1)	0.148
Positive	314 (37.4)	299 (35.6)	613 (36.5)	
Unknown	29 (3.5)	45 (5.4)	74 (4.4)	
AJCC stage				
I	380 (45.2)	406 (48.3)	786 (46.8)	0.363
II	312 (37.1)	303 (36.1)	615 (36.6)	
III	148 (17.6)	131 (15.6)	279 (16.6)	
ER status				
Negative	589 (70.1)	578 (68.8)	1,167 (69.5)	0.560
Positive	251 (29.9)	262 (31.2)	513 (30.5)	
PR status				
Negative	645 (76.8)	635 (75.6)	1,280 (76.2)	0.567
Positive	195 (23.2)	205 (24.4)	400 (23.8)	
HER2 status				
Negative	187 (22.3)	175 (20.8)	362 (21.5)	0.838
Positive	64 (7.6)	60 (7.1)	124 (7.4)	
Borderline	7 (0.8)	6 (0.7)	13 (0.8)	
Unknown	582 (69.3)	599 (71.3)	1,181 (70.3)	
Surgery type				
Mastectomy	377 (44.9)	354 (42.1)	731 (43.5)	0.527
BCS	462 (55.0)	485 (57.7)	947 (56.4)	
Unknown	1 (0.1)	1 (0.1)	2 (0.1)	
Radiation				
No	365 (43.5)	350 (41.7)	715 (42.6)	0.717
Yes	440 (52.4)	451 (53.7)	891 (53.0)	
Unknown	35 (4.2)	39 (4.6)	74 (4.4)	

AJCC = American Joint Committee on Cancer, BCS = breast conserving surgery, ER = estrogen receptor, PR = progesterone receptor, HER2 = human epidermal growth factor receptor 2, IDC = infiltrating ductal carcinoma, AAC = apocrine adenocarcinoma, IQR = interquartile range, LN = lymph node, NOS = no other specific, UD = undifferentiated.

^a^*P*-valuedetermined by Chi-square test comparing the matched AAC and IDC groups.

^b^Including American Indian/Alaskan native, and Asian/Pacific Islander, and others-unspecified.

^c^Including divorced, separated, single (never married), unmarried, domestic partner and widowed.

^d^Including grade 3 and undifferentiated.

### Subgroup analysis

In subgroup analysis of both OS and DSS, prognosis for patients with older age at diagnosis, white race and married status were worse in AAC histological type than in IDC type (AAC vs. IDC, for older age, HR = 1.252, 95% CI = 1.028–1.525 (OS); HR = 1.347, 95% CI = 1.045–1.736 (DSS); for white race, HR = 1.332, 95% CI = 1.078–1.646 (OS); HR = 1.392, 95% CI = 1.071–1.810 (DSS); for married status, HR = 1.363, 95% CI = 1.060–1.752 (OS); HR = 1.388, 95% CI = 1.028–1.872 (DSS)) (Table 4). Similarly, in subgroup of well differentiated diseases, AAC patients also showed poor prognosis than IDC patients (AAC vs. IDC, grade II tumors in OS analysis, HR = 1.391, 95% CI = 1.034–1.871; grade I tumors in DSS analysis, HR = 4.001, 95% CI = 1.286–12.450). Moreover, AAC patients also showed worse prognosis in subgroup of smaller tumors and positive LN (AAC vs. IDC, tumor size ≤ 2 cm, HR = 1.449, 95% CI = 1.081–1.942 (OS); HR = 1.838, 95% CI = 1.250–2.703 (DSS); positive LN, HR = 1.378, 95% CI = 1.099–1.726 (OS); HR = 1.448, 95% CI = 1.128–1.859 (DSS)). For different treatments, AAC patients were presented with worse prognosis in mastectomy and radiation recipients (AAC vs. IDC, mastectomy, HR = 1.319, 95% CI = 1.049–1.657(OS); HR = 1.340, 95% CI = 1.023–1.755 (DSS); radiation recipients, HR = 1.444, 95% CI = 1.121–1.861 (OS); HR = 1.534, 95% CI = 1.137–2.070 (DSS), respectively).

**Table 4:** Comparison of overall survival (OS) and disease-specific survival (DSS) between AAC vs IDC after subgroup analyses by univariate cox proportional hazard model

	OS		DSS	
	HR (95% CI)	*P*-Value^a^	HR (95% CI)	*P*-Value^a^
Age at diagnosis (years)				
18–49	1.222 (0.759–1.967)	0.409	1.397 (0.868–2.249)	0.169
50–79	1.252 (1.028–1.525)	0.026	1.347 (1.045–1.736)	0.022
Race				
White	1.332 (1.078–1.646)	0.008	1.392 (1.071–1.810)	0.014
Black	1.024 (0.636–1.649)	0.923	1.106 (0.641–1.907)	0.717
Others^b^	1.485 (0.860–2.563)	0.156	1.336 (0.666–2.678)	0.415
Marital status				
Married	1.363 (1.060–1.752)	0.016	1.388 (1.028–1.872)	0.032
Not married	1.261 (0.968–1.644)	0.086	1.320 (0.943–1.849)	0.106
Laterality				
Left	1.332 (0.987–1.798)	0.061	1.337 (1.052–1.700)	0.018
Right	1.328 (0.948–1.860)	0.099	1.223 (0.924–1.619)	0.160
Grade				
I	1.592 (0.662–3.828)	0.299	4.001 (1.286–12.450)	0.017
II	1.391 (1.034–1.871)	0.029	1.462 (0.978–2.183)	0.064
III + UD^c^	1.079 (0.844–1.380)	0.543	1.056 (0.795–1.402)	0.706
Tumor size (cm)				
≤ 2	1.449 (1.081–1.942)	0.013	1.838 (1.250–2.703)	0.002
2–5	0.994 (0.747–1.324)	0.970	0.834 (0.579–1.201)	0.330
> 5	0.955 (0.609–1.500)	0.842	1.012 (0.628–1.630)	0.961
LN status				
Negative	1.010 (0.721–1.414)	0.953	0.857 (0.507–1.448)	0.564
Positive	1.378 (1.099–1.726)	0.005	1.448 (1.128–1.859)	0.004
AJCC stage				
I	1.114 (0.746–1.664)	0.597	1.180 (0.613–2.271)	0.620
II	1.263 (0.942–1.692)	0.119	1.042 (0.704–1.543)	0.837
III	1.019 (0.765–1.357)	0.900	1.114 (0.825–1.504)	0.480
ER status				
Negative	0.852 (0.693–1.048)	0.129	0.709 (0.550–0.914)	0.008
Positive	1.121 (0.757–1.659)	0.568	1.402 (0.871–2.256)	0.164
PR status				
Negative	0.918 (0.751–1.122)	0.405	0.792 (0.618–1.014)	0.064
Positive	1.223 (0.789–1.896)	0.368	1.649 (0.976–2.787)	0.061
HER2 status				
Negative	0.867 (0.361–2.085)	0.750	1.044 (0.391–2.785)	0.931
Positive	1.384 (0.345–5.550)	0.647	1.065 (0.149–7.584)	0.950
Surgery type				
Mastectomy	1.319 (1.049–1.657)	0.018	1.340 (1.023–1.755)	0.034
BCS	1.129 (0.834–1.529)	0.431	1.147 (0.768–1.712)	0.504
Radiation				
No	1.142 (0.872–1.496)	0.334	1.179 (0.838–1.660)	0.345
Yes	1.444 (1.121–1.861)	0.005	1.534 (1.137–2.070)	0.005

AJCC = American Joint Committee on Cancer, AAC = apocrine adenocarcinoma, IDC = infiltrating ductal carcinoma, ER = estrogen receptor, PR = progesterone receptor, HER2 = human epidermal growth factor receptor 2, LN = lymph node, BCS = breast conserving surgery, HR = hazard ratio, CI = confidence interval, DSS = disease-specific survival, OS = overall survival.

^a^*P*-value was determined by univariate Cox proportional hazard regression model.

^b^Including American Indian/Alaskan native, and Asian/Pacific Islander, and others-unspecified.

^c^Including grade 3 and undifferentiated.

**Figure 3:**
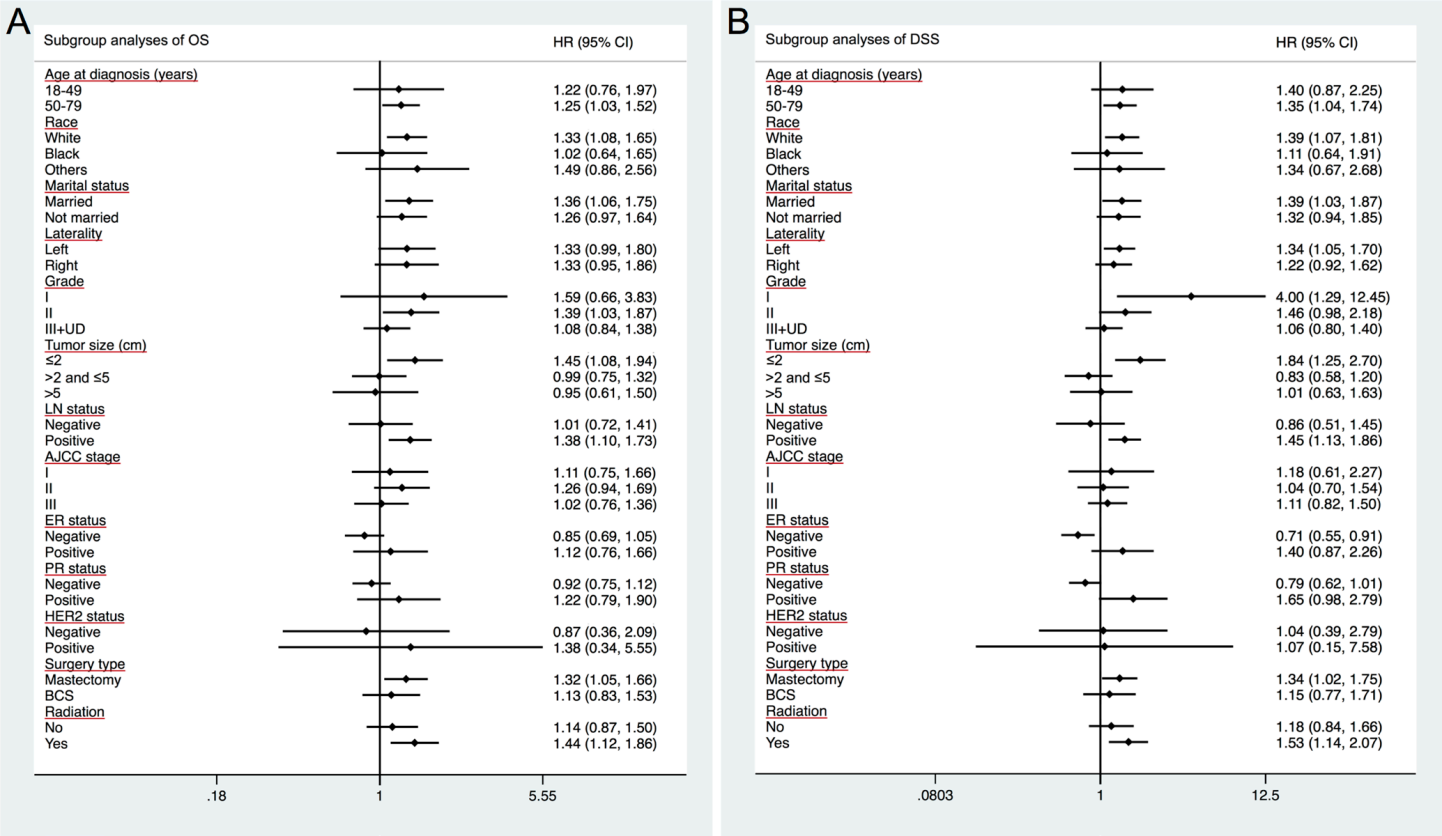
Forest plot of hazard ratios (HRs) for invasive apocrine adenocarcinoma (AAC) versus infiltrating ductal carcinoma (IDC) in the subgroup analysis The diamond on the X-axis indicates the HR and the 95% confident interval (CI) of each subgroup.

A forest plot of HRs that was used to illustrate the exploratory subgroup analyses suggested that in some subgroups, an AAC subtype was not a significant positive indicator of DSS or OS any longer (Figure 3). Specifically, HRs in different AJCC stage, ER status, PR status and HER2 status subgroups were not significantly different between AAC and IDC in subgroup analysis of OS. Similarly, HRs in different AJCC stage, PR status and HER2 status subgroups showed no significant difference between AAC and IDC for DSS. These results suggested that AJCC stage, ER status, PR status and HER2 status may be principal confounders for AAC prognosis.

### Stratification analysis with molecular subtype

Aiming to investigate the role of molecular subtype on breast cancer outcomes between AAC and IDC patients, a multivariate analysis stratified by molecular subtype was performed. As shown in Table 5, HR+/HER2- AAC patients showed poorer DSS than HR+/HER2- IDC patients (HR = 4.110, 95% CI: 1.026–16.465, *p* = 0.046). Triple-negative (TN)-AAC patients presented better DSS as well as OS than TN-IDC patients (HR = 0.203, 95% CI: 0.051–0.812, *p* = 0.024 (DSS); HR = 0.254, 95% CI: 0.082–0.787, *p* = 0.018 (OS), respectively).

**Table 5:** Comparison of disease-specific survival (DSS) and overall survival (OS) between AAC vs IDC after breast subtype analyses by univariate cox proportional hazard model

Subtype	DSS			OS		
	Events No./Sum No.	HR (95% CI)	*P*-Value^a^	Events No./Sum No.	HR (95% CI)	*P*-Value^a^
HR+/HER2-						
AAC	2/51	4.110 (1.026–16.465)	0.046	2/51	2.174 (0.543–8.702)	0.272
IDC	724/72,454	Reference		1,363/72,454	Reference	
HR+/HER2+						
AAC	0/22	–	–	0/22	–	–
IDC	129/12,268	Reference		231/12,268	Reference	
HR-/HER2+						
AAC	1/42	0.907 (0.127–6.479)	0.923	2/42	1.382 (0.343–5.562)	0.649
IDC	163/5,472	Reference		213/5,472	Reference	
Triple negative						
AAC	2/136	0.203 (0.051–0.812)	0.024	3/136	0.254 (0.082–0.787)	0.018
IDC	860/13,837	Reference		1,039/13,837	Reference	

AAC = apocrine adenocarcinoma, IDC = infiltrating ductal carcinoma, HER2 = human epidermal growth factor receptor 2, HR = hormone receptor, HR = hazard ratio, CI = confidence interval, DSS = disease-specific survival, OS = overall survival.

^a^*P*-value was determined by univariate Cox proportional hazard regression model.

## DISCUSSION

Apocrine carcinoma is a very rare and unique neoplasm of the breast, which is a morphologically distinct type from IDC. As it accounts for very small amount of all breast cancer, a large population is needed to obtain a sufficient number of patients with the relatively rare tumors. Therefore, we retrospectively investigated the clinicopathological and prognostic features of AAC in SEER database. Our findings indicated that AAC had unique clinicopathological characteristics and it tended to be a more aggressive type than IDC. Consistently, OS and DSS were both worse in ACC patients than in IDC patients with Kaplan-Meier analysis. Nevertheless, AAC patients presented similar survival outcomes in both OS and DSS to IDC after matching baseline characteristics. AAC histology was found not to be an independent prognostic factor after multivariate analysis in Cox proportional hazard model either. Further subgroup analysis indicated that AJCC stage, ER status, PR status and HER2 status may be principal confounders for AAC prognosis.

To our best knowledge, this study contained the largest number of patients compared to other published studies. We summarized both demographic and clinicopathological characteristics of AAC and found that this unique histological type was associated with an older age, lower proportion of white race, a larger tumor size, a higher grade, more positive LNs, an aggressive stage, lower ER and PR proportions, and higher HER2 amplification rates than that of IDC. As for the treatment strategies, AAC patients were less likely to be treated with breast-conserving surgery and adjuvant radiotherapy. These observations were partially in concordance with previous studies. Matsuo et al. reported that in patients with AAC, older age and postmenopausal status were observed more frequently than in those with IDC [9]. The study of Tanaka et al. and Dreyer et al. also confirmed that the patients with AAC were older [10, 11]. However, no significant difference with regard to menopausal status was observed between the two groups [10]. According to some literatures, AAC showed some non-aggressive behavior: axillary lymph node metastasis varies from < 1% to 4% [2]. Tanaka et al. also reported the proportion of AAC with LN metastasis and lymphatic invasion were significantly lower in the AAC patients than in the IDC patients [10]. In contrast, Dreyer et al. showed 7 out of 14 apocrine breast carcinomas with positive lymph node status [11] and Choi et al. reported the rage of lymph node metastasis was highest in the molecular apocrine type, although not statistically significant [21]. The inconsistence might be due to the heterogeneity of cohort since the study only involved very small number of patients.

Apocrine differentiation is generally inversely correlated with the expression of ER and PR but shows strong expression of the AR and HER2 or EGFR [3, 14, 22]. Moreover, expression of GCDFP-15 and CK20 is reported [23, 24]. In our present study, lower ER and PR proportions of AAC were reported. Consistently, several studies showed parallel results that the percentage of ER and PR receptor negativity was higher in the AAC group than in the IDC group [10, 17]. In addition, more positive HER2 status was shown in AAC patients than in IDC patients (7.6% vs. 6.8%, respectively; *p <* 0.001), which was consistent with others’ results [25]. Besides, some reported AAC was found very frequently in the triple-negative breast cancer (TNBC) group [17].

Most of previous studies revealed that AAC had a similar or more favorable prognosis when compared with IDC [8, 10, 15–18]. For example, Tanaka et al. reported 12% patients with AAC and 15% patients with IDC had experienced recurrences, while 5% patients with AAC and 8% patients with IDC died of recurrent breast cancer after a median follow-up period of 49 months. No significant differences in the relapse-free survival (*p* = 0.83) and overall survival (*p* = 0.75) rates were observed between the two groups [10]. Furthermore, Takeuchi et al. demonstrated the clinicopathological factors influencing 12-year survival rate were lymph node metastasis, lymphatic involvement and vascular involvement [17]. Although AAC and IDC had different clinicopathological characteristics, there was no difference in survival rates at 10 years after operation between AAC and non-AAC patients [17]. In the present study, we observed that OS and DSS were both worse in ACC patients than in IDC patients. Nevertheless, AAC patients presented similar survival outcomes in both OS and DSS to IDC when each AAC was matched with one IDC according to the most important prognostic parameters: age at diagnosis, pathological grade, tumor size, regional nodal status, ER status, PR status, HER2 status, treatment strategies and so on. Additionally, AAC histology was found not to be an independent prognostic factor after multivariate analysis in Cox proportional hazard model either. Interestingly, Nagao et al. reported AAC responded poorly to neoadjuvant chemotherapy (NAC). Despite their poor response to NAC treatment, patients with AAC was reported to have a good prognosis [15]. In accordance, Aoyagi et al. and Japaze et al. also reported that a significantly better outcome was observed in patients with AAC [12, 13].

In order to investigate the role of molecular subtype on prognoses between AAC and IDC patients, a multivariate analysis stratified by molecular subtype was performed as well. Interestingly, TN-AAC patients presented better DSS and OS outcomes than TN-IDC patients (*p* = 0.024). These results were in accordance with the findings of Iwase et al. and Choi et al. that AAC had better prognosis than non-AAC among TN breast cancers [21, 26]. Thus Iwase et al. suggested AAC should be regarded as different from the more common basal-like breast cancer [26]. Other studies suggested no significant differences if AAC compared with ductal carcinomas [20]. Notably, some of the histological subgroups contained insufficient numbers in order to draw firm conclusions [11].

The results of this study have several therapeutic implications. Since histological type was not an independent prognostic factor in the multivariate analysis, treatment guidelines do not need to be specified made based on this rare entity. Furthermore, since the subgroup analyses suggested that AJCC stage, ER status, PR status and HER2 status are the principal confounders for AAC prognosis; doctors should take more account of these prognostic indicators other than histological types.

Considering the similar prognostic outcomes of AAC to IDC, the existence of AAC of the breast as a distinct clinicopathological entity is debatable [27]. One strong reason for a designation of AAC is the identification of a subgroup of breast carcinomas that appear to have a unique response to androgen stimuli [28]. As an ER-positive breast cancer may benefit from estrogen deprivation treatment, some studies demonstrates that an AR-expressing AAC may respond to androgen deprivation [27]. It highlights the importance of further research elucidating the AR pathway in AAC, for which androgen represents the known steroid hormone stimulating tumor growth [29].

Inevitably, our study has several limitations. It has been widely accepted that AAC shows a characteristic steroid receptor profile: ER negative, PR negative, AR positive, and HER-2 or EGFR positive [25]. However, the status of HER-2 expression was not available until 2010 in SEER database. The status of AR and EGFR were not essential criteria for the diagnosis of AAC, therefore their expression were not recorded routinely in SEER database. Additionally, information regarding adjuvant chemotherapy and adjuvant endocrine therapy is absent from SEER database, which are both important prognostic factors for breast cancer. Other clinical parameters, like body mass index (BMI), age at first birth, family history of breast cancer as well as Eastern Cooperative Oncology Group (ECOG) scores need further investigation when these data are available in the future.

Collectively, we investigated a large population of patients with AAC and indicated that this rare histological type had unique clinicopathological characteristics and it tended to be a more aggressive type than that were observed in IDC patients. However, the worse prognosis was attenuated after adjusting for demographic and clinicopathological factors in the multivariate analysis. These results not only improve our understanding of the clinicopathological and prognostic features of this rare entity but also provide more convincing therapeutic guidelines for AAC of breast cancer patients.

## MATERIALS AND METHODS

### Ethics statement

We used National Cancer Institute's SEER data research files released in Nov 2015, which includes cancer registries covering 28% of the U.S. population. The data released by the SEER database do not require informed patient consent since cancer is a reportable disease in the United States. We obtained the permission to access the SEER database with the ID number 10444-Nov2015 via Internet access method. Our study was approved by the Ethical Committee of Shandong University.

### Patients selection

In order to identify eligible patients, we use the inclusion criteria as follows: female aged between 18 and 79, unilateral breast cancer, breast cancer (ICD-O-3 site code C50) as the first and only cancer diagnosis, diagnosis not obtained from a death certificate or autopsy, pathologic confirmation of infiltrating ductal carcinoma (IDC), not otherwise specified (ICD-O-3 8500/3) and apocrine adenocarcinoma (AAC) (ICD-O-3 8401/3)with invasion (behavior codeICD-O-3 malignant), known ER and PR statuses, American Joint Committee on Cancer (AJCC) stages I–III, and diagnosis from January 1, 2003 to December 31, 2013. We use the SEER*stat version 8.3.2 to generate a case-listing file. Finally, a total of 260,596 patients were included in our study. Of these patients, 840 were diagnosed with AAC and 259,756 with IDC.

Demographic characteristics included age at diagnosis, race, and marital status. We treated age at diagnosis as a binary variable classified into two groups: 18–49 years and 50–79 years. Tumor statistics included laterality, histological grade, tumor size, regional lymph node (LN) status, AJCC stage, ER status, PR status, and HER2 status. The evaluations of ER, PR as well as HER2 status were based on the guidelines from the American Society of Clinical Oncology (ASCO) and College of American Pathologists (CAP). Among those variables, known tumor size was treated as a categorical variable classified into the following groups: ≤ 2 cm, > 2 cm and ≤ 5 cm, or >5 cm. Due to the limitation of the SEER data files, HER2 status was only available until 2010 for both subtypes.

### Statistical analysis

Clinicopathological characteristics were compared between groups using Pearson's Chi-square test. Survival curves were generated using the Kaplan-Meier method, and differences between curves were analyzed with log-rank test. Multivariate Cox proportional hazard model was applied to estimate the association of covariates with overall survival (OS) and disease-specific survival (DSS). Subgroup analyses using univariate Cox proportional hazard model estimated the HRs of AAC versus IDC, and a forest plot was created to better present each prognostic factor's effect on OS and DSS. Hazard ratios (HRs) and 95% confidential intervals (CIs) were reported. These above statistical analyses were performed with SPSS version 18.0 (IBM SPSS Statistics, Chicago, IL, US).

To account for differences in baseline characteristics between groups, we matched 1 AAC patient with 1 IDC patient using the following predetermined factors: age at diagnosis, race, marital status, laterality, pathological grade, tumor size, regional LN status, AJCC stage, ER status, PR status, HER2 status, surgery type and radiation type. We utilized psmatch2 code in Stata version 12.0 (StataCorp, College Station, TX, US), which was designed for the propensity score matching method and to test the matching quality for the balance of the match. Two-sided *p-value* < 0.05 was considered as statistically significant.
